# Endoplasmic reticulum stress-mediated cell death in spinal cord injury: from molecular mechanisms to therapeutic applications

**DOI:** 10.3389/fcell.2026.1742297

**Published:** 2026-03-18

**Authors:** Wen-cong Zeng, Fang-jun Zeng

**Affiliations:** 1 Department of Spine Surgery, Ganzhou People’s Hospital, Ganzhou, Jiangxi, China; 2 Department of Spine Surgery, Ganzhou Hospital-Nanfang Hospital, Southern Medical University, Ganzhou, Jiangxi, China; 3 Department of Spine Surgery, Affiliated Ganzhou Hospital, Jiangxi Medical College, Nanchang University, Ganzhou, Jiangxi, China

**Keywords:** cell death, endoplasmic reticulum stress (ERS), spinal cord injury (SCI), therapeutic strategy, unfolded protein response (UPR)

## Abstract

Spinal cord injury (SCI) is a disabling central nervous system injury that can lead to permanent loss of motor and sensory function below the level of injury. Currently, symptoms are primarily alleviated and endogenous repair mechanisms are enhanced through surgical decompression, spinal fixation, hyperbaric oxygen therapy, and drug therapy, but these methods do not directly promote nerve regeneration and functional recovery. The endoplasmic reticulum is an important organelle that plays a crucial role in maintaining cellular homeostasis. Cell death is a significant pathological event in SCI, which further worsens the microenvironment at the injury site, leading to neurological dysfunction and affecting the clinical outcomes of patients. Adverse external stimuli can induce endoplasmic reticulum stress (ERS) in the body. ERS affects cellular homeostasis and fate by activating the unfolded protein response (UPR) and mainly participates in the pathological process of SCI through regulating autophagy, apoptosis, ferroptosis, necroptosis, and other cell death programs. Current evidence suggests that cell death induced by ERS may be an important pathological mechanism determining the prognosis and outcome of SCI. This article systematically reviews the research progress on ERS and UPR in the regulation of cell death in SCI. We focus on integrating the evidence and possible mechanisms linking ERS with four typical modes of cell death. Furthermore, we summarize advances in understanding the involvement of ERS/UPR in processes including inflammation amplification, mitochondrial dysfunction, oxidative stress, and nerve repair in SCI. On this basis, this article summarizes potential intervention targets and therapeutic strategies, aiming to provide a clearer integrative framework for understanding the pathological mechanisms of SCI and to offer reference directions for subsequent basic research and clinical translational studies.

## 1 Introduction

Spinal cord injury (SCI) is a highly destructive central nervous system trauma with a very high disability rate, which can lead to the loss of motor and sensory functions below the level of injury, as well as cause paraplegia or quadriplegia, putting patients in a predicament of lifelong disability (Mothe and Tator, 2012; Cofano et al., 2019). The main factors causing SCI include traffic accidents, sports-related injuries, and falls (Dukes et al., 2018; Li et al., 2020a). Based on pathological mechanisms and progression, SCI can be divided into primary injury and secondary injury. Primary injury is usually caused by external mechanical forces or trauma directly acting on the spine. This results in irreversible mechanical damage to spinal cord neurons (Anjum et al., 2020; Lukovic et al., 2015). Secondary injury involves pathological changes at the cellular and molecular levels in spinal cord tissue that follow primary injury, such as inflammation, oxidative stress, cell death, and mitochondrial dysfunction (Kim et al., 2017; Kwon et al., 2004; Al Mamun et al., 2021), leading to further mechanical and chemical damage to the spinal cord (Liu et al., 2021a). It is estimated that the global incidence of SCI ranges from 250,000 to 500,000 people each year (Khorasanizadeh et al., 2019), with the lifetime total cost for each SCI patient exceeding 3 million dollars (Katoh et al., 2019). This has a catastrophic impact on the quality of life of patients, and imposes a heavy burden on families and society (Zhou et al., 2017; Burns and O’connell, 2012). Currently, treatments for SCI mainly include surgical decompression and spinal stabilization, hyperbaric oxygen therapy, and drug therapy, all aiming to relieve spinal cord compression, stabilize the spine, and prevent complications (Karsy and Hawryluk, 2019). However, these traditional treatment methods have limited effects on the repair and functional recovery of damaged spinal cord neural tissue, making these treatments insufficient to achieve a fundamental breakthrough in nerve regeneration and functional reconstruction (Hutson and Di Giovanni, 2019). Therefore, in-depth exploration of the pathophysiological mechanisms after SCI is essential. Additionally, the search for more effective therapeutic targets and strategies has become a key issue that needs to be addressed in the field of neuroscience.

Endoplasmic reticulum stress (ERS), an important intracellular stress response, has received considerable attention in SCI research in recent years (Gow and Wrabetz, 2009). The endoplasmic reticulum is a key site for protein synthesis, folding, and modification in eukaryotic cells, as well as an important site for lipid synthesis. It plays a crucial role in maintaining the homeostasis of the intracellular environment (Zhang et al., 2022). When the spinal cord is injured, various stress factors such as ischemia, anoxia, oxidative stress, and calcium ion imbalance can cause endoplasmic reticulum dysfunction. This leads to the accumulation of unfolded or misfolded proteins in the endoplasmic reticulum lumen, initiating the endoplasmic reticulum stress response and triggering the unfolded protein response (UPR) (Oakes and Papa, 2015). In the short term, UPR acts as an adaptive response that enhances the cell’s ability to manage ERS by restoring ER homeostasis through protein processing, thereby promoting cell survival. However, prolonged ERS can exceed the adaptive capacity of UPR, resulting in terminal UPR activation and triggering cell death through various pathways (Szegezdi et al., 2006).

Increasing evidence suggests that endoplasmic reticulum stress-mediated cell death pathways play a significant role in the occurrence and development of numerous diseases (Martínez et al., 2018). These include neurodegenerative diseases such as Alzheimer’s disease (Lim et al., 2023) and Parkinson’s disease (Ren H. et al., 2021), as well as neurological injuries such as brain injury (Mi et al., 2023) and SCI (Zhou et al., 2020). These conditions are closely associated with ERS-related mechanisms. In the specific context of SCI, local ischemia and anoxia caused by trauma, together with the subsequent inflammatory response and surge of oxidative stress, lead to excessive activation of endoplasmic reticulum stress in spinal cord neurons and glial cells. This results in various forms of cell death, which significantly contribute to the deterioration of neurological function after SCI, exacerbating neuronal loss and worsening the condition (Sterner and Sterner, 2022; He et al., 2022a). By precisely regulating the endoplasmic reticulum stress response, it may be possible to alleviate secondary damage following SCI, promote neuronal survival and regeneration, and ultimately achieve effective recovery of spinal cord function, thereby improving patient prognosis and quality of life (Nomura et al., 2023). Therefore, in-depth research on the molecular mechanisms of endoplasmic reticulum stress in SCI is expected to provide new theoretical foundations and potential targets for developing innovative therapeutic strategies.

This article systematically reviews the molecular mechanisms of ERS-mediated cell death in SCI, integrating the latest research progress to explore the impact of ERS on the pathological process of SCI and the therapeutic significance of targeting ERS for SCI treatment. The aim is to provide theoretical support and future research directions for the clinical management of SCI.

## 2 Endoplasmic reticulum stress

### 2.1 Overview of ERS

The ER is a key organelle within eukaryotic cells. Its internal membrane system forms a complex network of tubular structures that are widely distributed in the cytoplasm. The ER connects externally with the cell membrane and internally with the nuclear membrane, resembling a “traffic hub” within the cell. It undertakes numerous important functions, such as protein and lipid synthesis and transport (Green and Reed, 1998; Lossi, 2022; Benyair et al., 2011). The ER has a sophisticated quality control system designed to ensure the correct folding and modification of proteins (Stefan et al., 2011). Under normal circumstances, the ER is rich in various chaperone proteins, glycosylation enzymes, and redox enzymes, which provide a suitable microenvironment for the folding of nascent peptide chains, promoting the folding of proteins into the correct conformation to perform their normal functions (Braakman and Bulleid, 2011). At the same time, the ER’s quality control system can identify and degrade improperly folded intermediates through endoplasmic reticulum-associated degradation (ERAD), thereby maintaining protein homeostasis within the ER (Meusser et al., 2005; Hwang and Qi, 2018). ERS refers to the disruption of the homeostatic balance of the ER under the influence of various harmful factors inside and outside the cell, leading to the accumulation of unfolded or misfolded proteins in the ER lumen, triggering a series of cellular stress responses (Hetz, 2012). However, when cells face adverse factors such as ischemia, hypoxia, oxidative stress, calcium ion imbalance, viral infections, and excessively rapid protein synthesis, the normal functions of the ER will be severely disrupted (Almanza et al., 2019). These stress factors can lead to a deterioration of the protein folding environment in the ER lumen, causing a sharp increase in the number of unfolded or misfolded proteins, exceeding the processing capacity of the ER. Consequently, cells will rapidly initiate the ERS response in an attempt to restore the homeostasis of the ER, thus ensuring cell survival. The ER stress response is primarily achieved through the activation of the UPR signaling pathway (Preissler and Ron, 2019). The UPR signaling pathway acts like an “emergency control center” for the cell. It is capable of sensing abnormalities in the protein folding state within the ER and transmitting signals to multiple targets in the nucleus and cytoplasm. This regulation affects the expression of a series of genes to enhance protein folding capacity, reduce protein synthesis, and accelerate the degradation of misfolded proteins. These processes help cells adapt to stress conditions and restore the normal physiological functions of the ER (Ren J. et al., 2021). If ERS persists and cannot be effectively alleviated, the cell will ultimately initiate apoptosis to sacrifice itself in order to maintain the overall homeostasis of the organism.

### 2.2 Triggers of ERS in SCI

#### 2.2.1 Impact of ischemia and hypoxia on spinal endoplasmic reticulum

**FIGURE 1:**
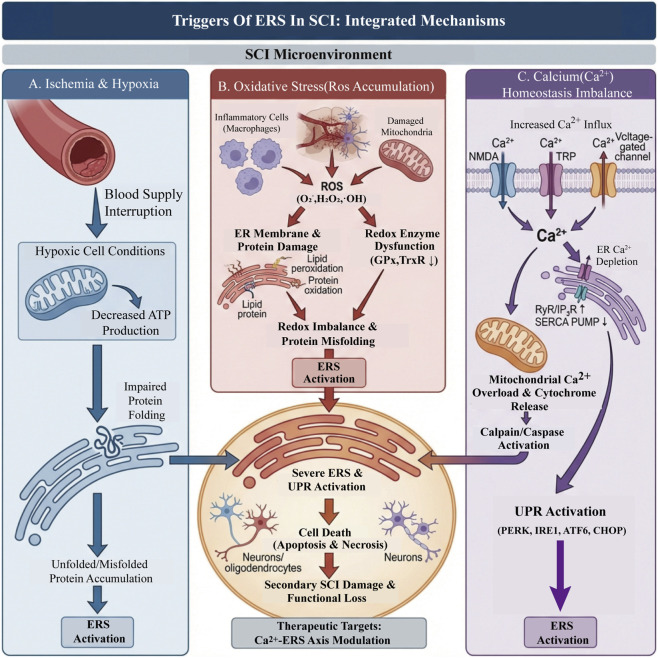
The integrated mechanisms triggering ERS in SCI microenvironment. **(A)** Ischemia and hypoxia reduce mitochondrial ATP production and impair protein folding, leading to accumulation of unfolded/misfolded proteins and ERS activation. **(B)** Oxidative stress from inflammatory cells and damaged mitochondria increases ROS, causing ER membrane lipid peroxidation, protein oxidation, redox enzyme dysfunction, and redox imbalance, which further promote ERS. **(C)** Disturbed Ca^2+^ homeostasis—with increased Ca^2+^ influx and ER Ca^2+^ depletion—induces mitochondrial Ca^2+^ overload, cytochrome-c release, and calpain/caspase activation, triggering the PERK/IRE1/ATF6-mediated UPR. Together these pathways drive severe ERS, neuronal/oligodendrocyte death, and secondary SCI damage, highlighting the Ca^2+^–ERS axis as a therapeutic target.

After SCI, the local tissue blood supply is interrupted, leading to ischemic and hypoxic conditions. Under these conditions, cellular energy metabolism is impaired, and ATP production decreases, which disrupts the energy-dependent protein folding processes in the ER (Anjum et al., 2020). For example, in a spinal cord ischemia-reperfusion injury model, studies have found that the protein folding capacity of the ER significantly decreases during the ischemic phase. This results in the accumulation of a large number of unfolded or misfolded proteins in the ER, thereby activating the ERS response (Luo et al., 2023). This activation of ERS not only affects the function of the ER itself, but also further exacerbates cellular damage and death through a series of signaling pathways (Zhao et al., 2019) (Figure 1).

#### 2.2.2 Disturbance of oxidative stress

Oxidative stress refers to the imbalance between oxidation and antioxidant systems in the body, leading to excessive production of reactive oxygen species (ROS). After SCI, various factors can trigger oxidative stress, such as inflammatory response and mitochondrial dysfunction (Islam et al., 2024). Consequently, as a key factor in the secondary injury after SCI, oxidative stress poses a serious threat to the homeostasis of the endoplasmic reticulum. Excessive ROS can damage the membrane structure and proteins of the endoplasmic reticulum, disrupting its redox balance and thereby interfering with the processes of protein folding and modification (Xie et al., 2024). Studies on the neurotoxicity of medical ozone in rats have shown that excessive ROS are generated when the body encounters external oxidative stimuli or when the intracellular redox balance is disrupted, including superoxide anion (O_2_^−^), hydrogen peroxide (H_2_O_2_), and hydroxyl radicals (·OH). The endoplasmic reticulum, which participates in intracellular redox reactions, becomes the primary target of ROS-induced damage. ROS can directly oxidize proteins in the endoplasmic reticulum lumen, damaging their secondary and tertiary structures and leading to protein misfolding. At the same time, ROS can interfere with the activity of key enzyme systems involved in redox regulation within the endoplasmic reticulum, such as glutathione peroxidase (GPx) and thioredoxin reductase (TrxR), causing an imbalance in its redox potential and further weakening protein folding capacity. Moreover, persistent oxidative stress can promote lipid peroxidation in the endoplasmic reticulum, damaging the integrity and fluidity of its membrane, affecting the function of related transport proteins and signal receptors on the membrane, ultimately activating the ERS signaling pathway and driving cells toward apoptosis (Li et al., 2018a). This indicates that oxidative stress is a major contributor to ERS, playing a key role in the pathological process of SCI.

#### 2.2.3 Chain reaction of calcium homeostasis imbalance

The ER is a key calcium reservoir within cells, tightly regulating the calcium concentration in the cytoplasm, which is crucial for normal physiological cell functions. The stability of its calcium content is essential for the correct folding of proteins and the normal function of the ER (Celik et al., 2023). In the context of SCI, primary mechanical impact and secondary cascades (ischemia/hypoxia, oxidative stress, membrane rupture, etc.) increase the permeability of the plasma membrane and cause various Ca^2+^channels (voltage-dependent Ca^2+^channels, NMDA receptors, TRP family channels) to open abnormally, inducing a sudden rise in cytoplasmic Ca^2+^. Simultaneously, the increase in Ca^2+^outflow from the ER mediated by RyR and IP_3_R channels of the ER coexists with impaired SERCA pump function, further disrupting Ca^2+^ homeostasis in the ER (Soni et al., 2023). ER calcium depletion and Ca^2+^overload synergistically trigger the UPR (PERK–eIF2α, IRE1–XBP1, ATF6) and downstream effects such as CHOP, which interfere with protein folding and lipid homeostasis, amplifying ERS (Celik et al., 2023; Soni et al., 2023). Excess Ca^2+^ also promotes mitochondrial Ca^2+^ uptake, ROS accumulation, and cytochrome c release through mitochondrial-endoplasmic reticulum contact sites (MAMs), thereby activating calcium-dependent proteases (such as calpain) and caspase cascades, leading to apoptosis/necroptosis of neurons and oligodendrocytes and driving the progression of secondary injury in SCI (Soni et al., 2023). Therefore, conducting combined interventions targeting the Ca^2+^ homeostasis–ERS axis in SCI may provide feasible targets for limiting secondary damage and promoting functional recovery.

## 3 Regulation of ERS and UPR

**FIGURE 2:**
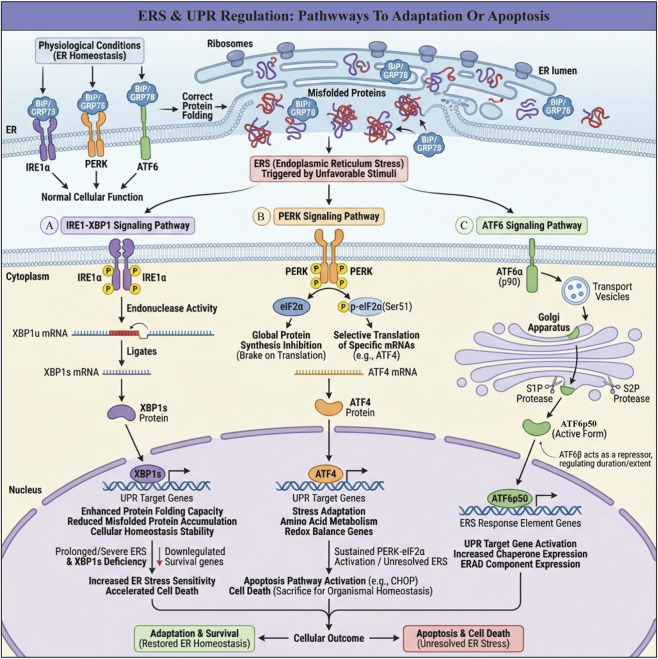
Three major pathways between adaptive survival and apoptotic death in cells after ERS activates UPR. **(A)** IRE1–XBP1 pathway: ERS activates IRE1, which splices XBP1 mRNA to XBP1s. Nuclear XBP1s induces chaperones and ERAD genes, enhancing protein folding/clearance and restoring ER homeostasis; persistent stress reduces this protection and increases cell death sensitivity. **(B)** PERK–eIF2α–ATF4 pathway: PERK-mediated eIF2α phosphorylation globally suppresses translation but favors ATF4 synthesis. ATF4 initially drives adaptive and antioxidant genes; under prolonged, unresolved stress it upregulates CHOP and other pro-apoptotic genes, triggering apoptosis. **(C)** ATF6 pathway: ERS causes ATF6 to move to the Golgi, where it is cleaved into an active fragment that enters the nucleus and induces chaperone and ERAD genes, boosting folding capacity.

In maintaining normal cell homeostasis, molecular chaperones in the endoplasmic reticulum lumen assist newly synthesized proteins in achieving the correct three-dimensional conformation, thus enabling them to perform their normal functions (Ellgaard and Helenius, 2001). When cells are stimulated by adverse external factors, ERS can be triggered, activating the UPR (Preissler and Ron, 2019). UPR activation transmits stress signals from the ER to the nucleus to regulate related genes, restoring ER homeostasis and recovering normal physiological functions. However, continuous stress stimuli during ERS can lead to cell death (Cybulsky, 2017). The ER contains three transmembrane protein receptors: inositol-requiring enzyme 1α (IRE1α), protein kinase RNA-like endoplasmic reticulum kinase (PERK), and activating transcription factor 6 (ATF6) (Almanza et al., 2019). Under physiological conditions, these transmembrane receptors are maintained in an inactive state by binding to molecular chaperones, immunoglobulin heavy chain binding protein (BiP), or glucose-regulated protein 78 (GRP78). When ERS occurs, BiP or GRP78 dissociates from the transmembrane receptors; this initiates three protein-mediated signaling pathways, activates related downstream signals, and ultimately regulates the expression of related genes (Ron and Walter, 2007; Bertolotti et al., 2000) (Figure 2).

### 3.1 IRE1-XBP1 pathway

Inositol-Requiring Enzyme 1α (IRE1α) is an evolutionarily conserved ER stress sensor and an I-type transmembrane protein in the endoplasmic reticulum of all eukaryotes, including yeast and humans, playing a key role in maintaining endoplasmic reticulum homeostasis (Adams et al., 2019). Under physiological conditions, IRE1α exists as a monomer on the endoplasmic reticulum membrane, with its luminal domain tightly bound to BiP/GRP78, in an inactive state (Bertolotti et al., 2000). During ERS, BiP/GRP78 dissociates from IRE1α, allowing IRE1α to homodimerize and autophosphorylate. Phosphorylated IRE1α exhibits kinase and endonuclease activities; its endonuclease domain cleaves the mRNA of its target gene, X-box binding protein 1 (XBP1), generating the active spliced form, XBP1s (Raymundo et al., 2020; Malhi and Kaufman, 2011). XBP1s, as a transcription factor, regulates the expression of a series of genes that help cells enhance protein folding capacity, reduce the accumulation of misfolded proteins, and promote intracellular homeostasis (Nishida et al., 2017). The absence of XBP1 has a significant impact on cells, especially under ER stress conditions. Studies have shown that loss of XBP1 decreases the ability of cells to adapt to ER stress, thereby exacerbating cell death. For example, the absence of XBP1 in mouse models results in reduced white matter protection after SCI, affecting functional recovery (Xu et al., 2024). Furthermore, loss of XBP1 leads to downregulation of a series of genes related to cell survival, increasing cell sensitivity to ER stress and accelerating cell death (Flores-santibañez et al., 2023).

### 3.2 Protein kinase RNA-Like endoplasmic reticulum kinase

Protein kinase RNA-like endoplasmic reticulum kinase (PERK) is also an I-type transmembrane protein on the endoplasmic reticulum membrane, structurally highly similar to IRE1α (Kopp et al., 2019). In the absence of stress stimuli, it remains stable by binding to BiP. After ERS occurs, PERK acts as an awakened guardian, quickly sensing the disruption of the protein folding environment in the endoplasmic reticulum lumen. PERK separates from BiP and undergoes oligomerization and trans-autophosphorylation to become activated. The activated PERK targets eukaryotic initiation factor 2α (eIF2α), catalyzing the phosphorylation of the serine residue at position 51. This phosphorylation modification acts as a brake signal, inhibiting the activity of eIF2α and significantly reducing global protein synthesis to maintain cell survival, (Harding et al., 2003). Phosphorylation of eIF2α not only inhibits protein synthesis but also exerts intricate regulatory effects. On one hand, phosphorylated eIF2α promotes the preferential translation of specific mRNAs, such as activating transcription factor 4 (ATF4). As a key transcriptional regulatory factor, ATF4 can activate the expression of a series of genes related to cellular stress adaptation, amino acid metabolism, and redox balance, helping cells survive in harsh environments (Hetz et al., 2020). On the other hand, sustained activation of the PERK-eIF2α pathway may also trigger the initiation of apoptosis to some extent (Zheng et al., 2014). When ERS cannot be effectively alleviated, the excessive accumulation of stress signals will drive cells toward apoptosis, a process that helps maintain overall homeostasis by removing irreparably damaged cells (Donnelly et al., 2013).

### 3.3 Activate transcription factor 6 signaling pathway

Transcription factor 6 (ATF6) is a type II transmembrane protein with a large structural domain containing a bZIP transcription factor dimerization domain, which can function as a transcription factor (Li et al., 2024). There are two mammalian homologs of ATF6, ATF6α and ATF6β, which contain different transcriptional activation domains. It is believed that ATF6β may act as a repressor, regulating the duration and extent of ATF6α-mediated gene regulation in the UPR (Thuerauf et al., 2004). Both ATF6α and ATF6β are anchored to the endoplasmic reticulum membrane in an inactive precursor form. During ERS, ATF6α dissociates from GRP78 (also known as BiP), a chaperone involved in the processing and folding of unfolded proteins. This release allows ATF6α to be transported to the cis-Golgi apparatus (Yamamoto et al., 2007). In the Golgi apparatus, full-length ATF6α is sequentially cleaved by two Golgi-resident processing enzymes into active ATF6α (ATF6p50), namely, site 1 protease (S1P)/MBTPS1 and site 2 protease (S2P)/MBTPS2. After proteolytic cleavage, active ATF6α (ATF6p50) is transported to the nucleus, where it activates UPR target genes. Additionally, ATF6p50 can bind to genes associated with ERS response elements and regulate their transcription and expression (Yamamoto et al., 2007; Zhang and Kaufman, 2008).

## 4 ERS mediated cell death in SCI

**FIGURE 3:**
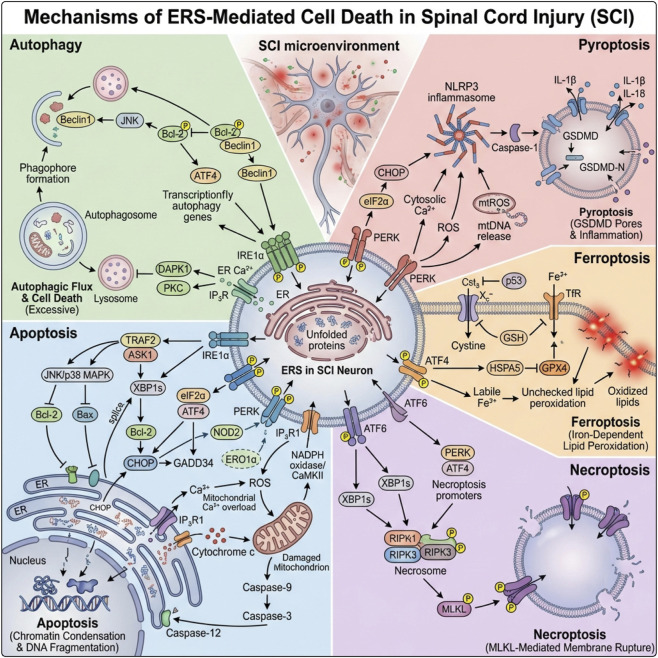
Multiple cell death patterns driven by ERS in SCI. In the center, unresolved ERS/UPR in injured neurons (PERK, IRE1α, ATF6) is triggered by unfolded proteins, Ca^2+^ dysregulation and oxidative stress from the SCI microenvironment. Upper left: ERS activates ATF4- and IRE1α-dependent signaling that modulates Beclin1/Bcl-2 and ER–Ca^2+^ release, shifting adaptive autophagy toward excessive autophagic cell death. Lower left: CHOP induction, Bcl-2 downregulation, mitochondrial Ca^2+^ overload and cytochrome-c release activate caspase-9/3, leading to apoptosis. Upper right: ERS-related ROS, mtDNA release and cytosolic Ca^2+^ promote NLRP3 inflammasome assembly and caspase-1 activation, driving GSDMD-mediated membrane pore formation and pyroptosis with IL-1β/IL-18 release. Middle right: ATF4 and p53 reprogram iron and lipid metabolism; GPX4 inhibition and unchecked lipid peroxidation culminate in iron-dependent ferroptosis. Lower right: PERK/ATF4 signaling upregulates RIPK1/RIPK3–MLKL, forming the necrosome and causing necroptotic membrane rupture. Together, ERS functions as a central hub linking autophagy, apoptosis, pyroptosis, ferroptosis and necroptosis, and represents a key therapeutic target in secondary SCI.

ERS can initiate UPR to restore cellular protein homeostasis and promote cell survival. However, when ERS is severe or persistent and cannot be compensated, UPR may fail, leading to different modes of cell death, such as autophagy, apoptosis, pyroptosis, ferroptosis, and necroptosis (Figure 3).

### 4.1 Autophagy

Autophagy is a highly conserved process for maintaining intracellular homeostasis and adapting to stress. It primarily degrades and recycles damaged organelles and abnormal proteins through the autophagosome-lysosome pathway to support energy supply, maintain metabolic homeostasis, and promote cell survival. It is important to emphasize that autophagy itself is typically regarded as a cell protective mechanism rather than a form of PCD. Autophagy can shift from an “adaptive response” to promoting damage and participate in or accompany the cell death process under specific circumstances—sometimes described as autophagy-related/autophagic cell death—only when it is excessively activated for an extended period, critical survival signals are impaired, or autophagic flux is obstructed, leading to degradation failure and accumulation of harmful substances within the cell (Klionsky and Emr, 2000; Shin et al., 2016). There are three types of autophagy, which serve as key self-protection and material recycling mechanisms within the cell: macro-autophagy, micro-autophagy, and chaperone-mediated autophagy. Among them, macro-autophagy is the most extensively studied type. Existing research indicates a close relationship between ERS and autophagy, particularly because ERS can regulate the initiation and flux of autophagy through UPR-related pathways, thereby affecting cell fate and the inflammatory microenvironment in SCI (Yorimitsu et al., 2006).

If ERS continues to intensify and cannot be alleviated, excessively activated autophagy may become detrimental to the cell, contributing to cell death. In the process of ER stress-induced autophagy, the three UPR sensors—Ire1α, PERK, and ATF6—regulate autophagy through their respective pathways. IRE1 can activate apoptosis signal-regulating kinase 1 (ASK1) through tumor necrosis factor receptor-associated factor 2 (TRAF2), thereby triggering c-Jun N-terminal kinase (c-JNK); JNK mediates the phosphorylation of Bcl-2, disrupting the interaction between Bcl-2 and Beclin1, thus inducing autophagy (Liu C. et al., 2020; Fernández et al., 2015). The PERK signaling pathway also plays a crucial role in ERS-induced autophagy. Both PERK and ATF4 are essential for this process (Luhr et al., 2019). During ERS regulation of autophagy gene expression, ATF4 can directly bind to and upregulate multiple autophagy gene promoters to initiate autophagy (B’chir et al., 2013). The ATF6 signaling pathway is a key pathway in the UPR process. ATF6 is involved in ER stress-induced autophagy by promoting autophagy through upregulating the expression of CHOP (Motawi et al., 2022). Additionally, Ca^2+^ plays an important role in ER stress-induced autophagy. ERS can activate the expression of Ca^2+^ release channels on inositol 1,4,5-trisphosphate receptors (IP3R), thereby mediating the release of ER-Ca^2+^. The release of Ca^2+^ from the endoplasmic reticulum participates in the regulation of autophagy by activating calcium-dependent kinases such as death-associated protein kinase 1 (DAPK1) and protein kinase C (PKC) (Sakaki and Kaufman, 2008; Høyer-Hansen et al., 2007).

### 4.2 Apoptosis

Apoptosis is a process of cell death that occurs through the activation of caspase cascades under the strict regulation of apoptotic genes, and it is also the most common form of PCD (Tang et al., 2019; Maiuri et al., 2007). Its characteristics include DNA degradation, nuclear condensation and fragmentation, and the formation of apoptotic bodies (Garrido et al., 2006). ERS has been shown to be another regulatory pathway for apoptosis, when ERS occurs, three transmembrane protein receptors on the ER membrane are activated, leading to apoptosis (Dong et al., 2019).

As one of the key pathways mediating cell death due to ERS, apoptosis plays a central role in the fate decision of neurons after SCI. When ERS is triggered and cannot be alleviated, UPR-related signaling pathways activate a series of pro-apoptotic molecules. Among them, CHOP (C/EBP homologous protein) is a key “executor” of the apoptotic process. IRE1α plays a crucial role in linking ERS and apoptosis. When ERS occurs, activated IRE1α interacts with CHOP through XBP1. It also activates downstream c-JNK or p38 mitogen-activated protein kinase (p38-MAPK) gene expression via the TRAF2-ASK pathway. These actions inhibit the anti-apoptotic protein Bcl-2 and promote the translocation of the pro-apoptotic protein Bax to the mitochondrial membrane, thereby leading to apoptosis (Wang and Ron, 1996; Siwecka et al., 2021). In SCI models, studies have found that early after injury, CHOP expression in spinal cord neurons is significantly upregulated and closely positively correlated with the number of apoptotic neurons. CHOP inhibits the activity of anti-apoptotic proteins Bcl-2 and Bcl-xL, disrupting the balance between apoptosis and anti-apoptosis within the cell, making it easier for the cell to undergo apoptotic outcomes (Urano et al., 2000). PERK promotes eIF2α phosphorylation after dimerization and trans-autophosphorylation, subsequently inducing ATF4 synthesis and translocation. After ATF4 enters the nucleus, it upregulates the expression of CHOP and downstream factor GADD34. This alters the expression of apoptotic proteins such as Bax and Bcl-2, thereby causing apoptosis (Toth et al., 2007; Li Y. et al., 2019). Similarly, ATF6 can also promote apoptosis through the CHOP pathway. Nucleotide-binding oligomerization domain protein (2NOD2) interacts with ATF6 and acts as a negative regulator of ATF6 activation and its downstream target molecule CHOP, thus inhibiting ERS-induced apoptosis (Kwon et al., 2020; Huang L. et al., 2021). In addition to the above factors, the Ca^2+^ released intracellularly due to ERS can also lead to apoptosis (Wang et al., 2021). ERO1α activates IP3R1, which mediates the release of ER Ca^2+^, allowing Ca^2+^ to enter the cytoplasm. This subsequently activates CaMKII and NADPH oxidase on the cell membrane, which then triggers the release of cytochrome C from the mitochondria, promoting the generation of ROS and apoptotic bodies, thereby initiating caspase-9 mediated apoptosis (Munoz-Pinedo et al., 2006; Means and Katz, 2022; Morris et al., 2021).

Caspase-12 is an ERS-specific apoptotic protease that is normally localized in an inactive zymogen form on the endoplasmic reticulum membrane (Nakagawa et al., 2000). When ERS occurs—particularly due to an imbalance in endoplasmic reticulum calcium homeostasis accompanied by damage such as lipid peroxidation—Caspase-12 is activated. The activated Caspase-12 then translocates from the endoplasmic reticulum membrane to the cytoplasm, where it directly cleaves and activates downstream executioner caspases, including Caspase-9 and Caspase-3. This activation triggers a series of proteolytic reactions leading to typical apoptotic features such as cytoskeletal disassembly and DNA fragmentation. These events promote the rapid progression of apoptosis and exacerbate cell death, thereby impairing neurological function (W et al., 2020; Gu et al., 2016).

### 4.3 Pyroptosis

Pyroptosis is a type of pro-inflammatory PCD mediated by inflammatory caspases, characterized by pore formation mediated by gasdermin family proteins. The classical pyroptosis pathway is primarily driven by caspase-1; after the assembly of the inflammasome, caspase-1 is activated. It then cleaves GSDMD and promotes the maturation and release of IL-1β/IL-18 (Liu and Sun, 2019; Galluzzi et al., 2015; Fink and Cookson, 2006). The non-classical pyroptosis pathway is mediated by caspase-4/5 (human) or caspase-11 (mouse), which directly senses cytoplasmic LPS and cleaves GSDMD, often subsequently leading to activation of the NLRP3-caspase-1 axis, thereby amplifying the inflammatory response. In addition to the two major pathways mentioned above, under specific pathological conditions, caspase-3 or caspase-8 can cleave GSDME or, in some cases, other gasdermin members, inducing membrane pore formation and pro-inflammatory cell lysis phenotypes similar to pyroptosis (Lacey et al., 2018; Wang Y. et al., 2017). The morphological characteristics of pyroptosis, which significantly differ from the molecular execution mechanisms of apoptosis and other forms of programmed cell death, typically manifest as a rapid loss of cell membrane integrity, cell swelling, and membrane pore formation, releasing inflammatory mediators (Fischer et al., 2021; Yu et al., 2021).

When ERS occurs, especially under complex and adverse pathological conditions following SCI, intracellular inflammasomes are abnormally activated, becoming key triggers for the initiation of pyroptosis. Among these inflammasomes, the NOD-like receptor pyrin domain containing 3 (NLRP3), inflammasome is one of the most extensively studied members. In SCI models, researchers found that NLRP3 expression in spinal cord tissue was significantly upregulated early after injury and positively correlated with the degree of local inflammatory cell infiltration and the expression of pyroptosis markers. A series of adverse events—including oxidative stress resulting from ERS, calcium homeostasis imbalance, and mitochondrial damage—can serve as danger signals for NLRP3 inflammasome activation (Gu and Liu, 2025). Studies have found that IRE1α can promote the cleavage of GSDMD protein, and exacerbate pyroptosis by activating the NLRP3 pathway (Ke et al., 2020). The PERK and eIF2α pathways can induce the expression of CHOP, which, when upregulated, promotes NLRP3 production and leads to activation of NLRP3 inflammasomes, thereby inducing pyroptosis (Li et al., 2022). Additionally, the increase in cytosolic calcium concentration caused by ER calcium release can synergize with ROS to enhance NLRP3 activity. Substances released from damaged mitochondria, such as mitochondrial DNA and mitochondrial ROS (mtROS), further promote the activation of NLRP3 inflammasomes and induce pyroptosis (Li et al., 2023).

### 4.4 Ferroptosis

Ferroptosis is a form of cell death caused by the accumulation of iron-dependent lipid peroxides, characterized mainly by abnormal iron metabolism and lipid peroxidation within the cells (Dixon et al., 2012). After SCI, the disruption of the local microenvironment triggers a series of chain reactions, creating a favorable environment for ferroptosis. ERS plays a key role and is closely connected to ferroptosis through multiple pathways (Wei et al., 2021). ERS can lead to an imbalance in intracellular iron metabolism. Under normal physiological conditions, intracellular iron homeostasis is precisely regulated by transferrin receptor (TfR), ferritin, and other related proteins. However, under stress conditions, the activation of ERS-related signaling pathways, such as the PERK pathway, can upregulate the expression of TfR, thereby enhancing the cell’s ability to uptake iron ions. Additionally, ERS can inhibit the activity of System Xc–via p53-mediated pathways, reducing the synthesis of glutathione (GSH) and ultimately promoting ferroptosis (Zheng et al., 2022). Furthermore, the activation of ATF4 induces HSPA5 to bind with GPX4 to form a complex, which inhibits GSH-glutathione peroxidase 4 (GPX4) degradation and thus protects cells from ferroptosis (Zheng et al., 2022). In addition, ERS also affects the antioxidant defense system, especially the GPX4 axis. ERS can interfere with the metabolic synthesis of GSH, reducing its production; furthermore, it inhibits the activity of GPX4, impairing the cell’s ability to effectively clear accumulated lipid peroxides (Xie et al., 2016). The lipid peroxidation reaction, catalyzed by iron ions, occurs through the classical Fenton reaction or Fenton-like reactions. These ROS act as harmful agents, indiscriminately attacking unsaturated fatty acids in the cell membrane, triggering a lipid peroxidation chain reaction that damages the integrity and fluidity of the cell membrane, ultimately leading to cell swelling, rupture, and ferroptosis (Ho et al., 2021).

### 4.5 Necroptosis

Necroptosis refers to a form of programmed cell death mediated by genetically regulated processes involving receptor-interacting serine/threonine protein kinase 1/3 (RIPK1/3) through receptor-mediated signaling (Degterev et al., 2005; Zhou et al., 2022). Necroptosis can be induced by various stimuli, including TNF, FasL, and TRAIL (Vanlan et al., 2012). The execution of necroptosis involves the coordinated action of multiple key molecules and signaling pathways. RIPK1 and RIPK3 act as core regulatory factors in ERS-induced necroptosis (Mohammed Thangameeran et al., 2020). When cells are subjected to lethal stimuli such as ERS, RIPK1 undergoes autophosphorylation and interacts with RIPK3 to form a necrosome complex. This complex then recruits and phosphorylates mixed lineage kinase domain-like protein (MLKL). Phosphorylated MLKL undergoes a conformational change, polymerizes from monomers into oligomers, and translocates to the cell membrane. This leads to disruption of membrane integrity, cell swelling, and rupture, ultimately triggering necroptosis (Fan et al., 2015). There is a close and complex intrinsic relationship between necroptosis and ERS, profoundly affecting the pathological process and neural repair outcomes following SCI (Silva et al., 2022). Under normal physiological conditions, precise regulatory mechanisms within cells maintain homeostasis and survival. However, after SCI, ERS is strongly induced, and multiple factors converge to drive cells toward necroptosis (Fan et al., 2015). In this process, ERS-related signaling pathways and necroptosis pathways interact and communicate, forming a complex regulatory network. For example, activation of the IRE1α-XBP1 pathway produces XBP1s, which participates in the adaptive regulation of ERS and indirectly influences necroptosis by regulating the expression of certain anti-apoptotic proteins (Wang S. et al., 2017). Sustained activation of the PERK-eIF2α pathway promotes increased expression of ATF4, which, besides its role in ERS-mediated apoptosis regulation, can bind to promoter regions of necroptosis-related genes, regulating their transcriptional activity and affecting necroptosis (Iurlaro and Muñoz-Pinedo, 2016). Upon ATF6 activation, its released active fragments enter the nucleus to regulate the expression of various ERS-related genes and may also indirectly regulate necroptosis-related molecules, although the specific mechanisms require further investigation (Iurlaro and Muñoz-Pinedo, 2016).

## 5 Effects of ERS on other pathological processes of SCI

### 5.1 Oxidative stress

Oxidative stress refers to the excessive production of ROS and reactive nitrogen species (RNS) when the body is subjected to various external or internal stimuli, leading to an imbalance between the oxidative system and the antioxidant system due to reduced antioxidant system function, resulting in cell damage (Pizzino et al., 2024). Under normal circumstances, the redox system within cells is in dynamic equilibrium, with stable production and clearance of ROS. However, after SCI, the local ischemic and hypoxic environment rapidly triggers a series of biochemical reactions, resulting in a large amount of ROS generation (Xiao et al., 2024). On one hand, during the ischemia-reperfusion process, the dysfunction of the mitochondrial electron transport chain increases electron leakage, leading to an explosive production of superoxide anion (O_2_^−^) and other ROS (Yu et al., 2023); on the other hand, the upregulation of xanthine oxidase, NADPH oxidase, and other activities in damaged cells further catalyzes the generation of ROS (Weng et al., 2024).

There is a close bidirectional regulatory relationship between ERS and oxidative stress. When ERS occurs, the homeostasis within the endoplasmic reticulum is disrupted, and the accumulation of unfolded or misfolded proteins can directly lead to an increase in ROS production (Tang et al., 2022). For example, the IRE1α-XBP1 pathway and PERK-eIF2α pathway activated by ERS interfere with the metabolic processes within cells, causing abnormal supply of the electron donor NADPH for the electron transport chain, thereby promoting the mitochondria to produce more ROS. At the same time, ROS, as a signaling molecule, can further exacerbate the ERS response (Malhotra et al., 2007). High concentrations of ROS can oxidatively modify proteins within the ER lumen, making them more prone to misfolding, further increasing the protein load on the ER; ROS can also attack the lipid components of the ER membrane, damaging the structural integrity of the ER and affecting its normal function, forming a vicious cycle that continuously elevates the levels of oxidative stress and ERS within cells (Zhao and Ackerman, 2006).

In the pathological environment following SCI, oxidative stress and ERS work synergistically, delivering multiple blows to nerve cells. The massive accumulation of ROS can directly damage the biomacromolecules of nerve cells, such as causing lipid peroxidation of cell membranes, damaging mitochondrial DNA and proteins, and leading to oxidative modifications of nucleic acids, thereby destroying the structural and functional integrity of cells, ultimately inducing apoptosis or necrosis (Lee et al., 2014); in addition, the inflammatory signaling pathways activated by both oxidative stress and ERS promote the release of a large number of inflammatory factors, attracting immune cell infiltration, further exacerbating the local inflammatory response, forming a positive feedback loop that continuously amplifies the damage signal, severely hindering nerve repair and regeneration after SCI (Ohri et al., 2011). Therefore, breaking the vicious cycle between oxidative stress and ERS has become one of the key links in intervening in the pathological process of SCI and promoting the recovery of nerve function.

### 5.2 Inflammatory response

Inflammation not only has a clearing effect on necrotic tissues and cells, but persistent inflammatory responses can exacerbate tissue and organ damage, leading to cell death (Liu et al., 2021b; Jridi et al., 2020). After SCI, local tissue is damaged, the blood-spinal cord barrier is disrupted; leukocytes and inflammatory cytokines in the blood rapidly flood into the injury site, activating a series of inflammatory signaling pathways and inducing a strong inflammatory response (Kigerl et al., 2014; Lehnardt, 2010). The different stages after SCI exhibit different manifestations and effects of the inflammatory response.

There is a complex molecular relationship between ERS and inflammatory responses. ERS can promote the release of inflammatory cytokines by activating multiple signaling pathways, including the IRE1α-XBP1, PERK-eIF2α, and ATF6 pathways, which can upregulate the expression of inflammatory cytokines such as TNF-α, IL-6, and IL-1β (Li W. et al., 2020). These inflammatory cytokines can not only directly damage neuronal cells but also further activate immune cells, such as macrophages and microglia, causing them to release more inflammatory cytokines, forming a positive feedback loop that exacerbates the inflammatory response and leads to continuous expansion of neural tissue damage (Adolph et al., 2012). Moreover, the inflammatory response can affect the degree of ERS. Free radicals such as ROS and NO, as well as cytokines in the inflammatory microenvironment, can interfere with the normal function of the ER, promoting abnormal protein folding within the ER and exacerbating ERS (Hasnain et al., 2012). For example, ROS can oxidize proteins in the ER lumen, making them more prone to misfolding and increasing the protein load on the endoplasmic reticulum. NO can interfere with the activity of ER-related enzymes, disrupting the processes of protein modification and folding, further disrupting the homeostasis of the ER (Niella et al., 2024).

### 5.3 Blood spinal barrier integrity

The blood-spinal cord barrier (BSCB) is a highly specialized spinal cord endothelial structure that serves as a critical defense line between spinal cord tissue and blood circulation, playing an indispensable role in maintaining the homeostasis of the spinal cord environment (Sweeney et al., 2018). It is composed of various components, including spinal microvascular endothelial cells, the basement membrane, astrocytic end-feet, and pericytes, which work closely together to strictly regulate the entry and exit of substances in the blood, thereby providing a stable and suitable microenvironment for spinal neurons to ensure their normal function (Zhao et al., 2015).

After SCI, ERS significantly disrupts the integrity of the BSCB (Hu et al., 2024). The inflammatory response triggered by ERS is a key factor contributing to BSCB disruption. ERS promotes the release of numerous inflammatory factors such as TNF-α and IL-6, which directly damage endothelial cells of the BSCB by downregulating the expression of tight junction proteins (such as occludin and claudin-5) and causing their aberrant distribution. This results in increased gaps between endothelial cells and a marked increase in barrier permeability (Huang X. et al., 2021). Moreover, inflammatory factors activate signaling pathways within endothelial cells, such as the nuclear factor-κB (NF-κB) pathway, further upregulating the expression of adhesion molecules (such as ICAM-1 and VCAM-1), which facilitates leukocyte adhesion and transmigration across the endothelium, thereby exacerbating BSCB damage. Consequently, harmful substances in the blood—including immune cells, inflammatory mediators, and large molecular proteins—can infiltrate spinal cord tissue, establishing a vicious cycle that perpetuates and amplifies tissue damage (Xin et al., 2023).

**FIGURE 4:**
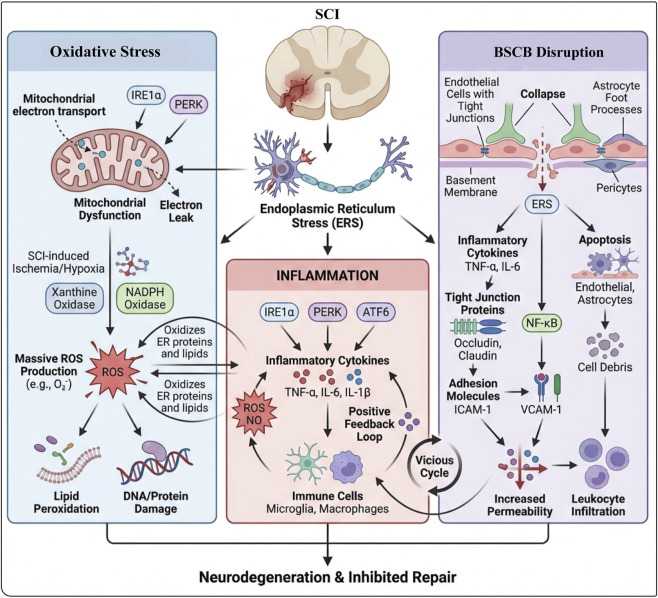
The vicious cycle linking oxidative stress, ERS, and BSCB disruption after SCI. Left: SCI-induced ischemia/hypoxia and activation of mitochondrial and NADPH oxidases cause massive ROS production, driving lipid peroxidation, DNA/protein damage, and oxidation of ER proteins and lipids, thereby triggering ERS. Center: ERS pathways (IRE1α, PERK, ATF6) amplify inflammation, with microglia and macrophages releasing TNF-α, IL-6, and IL-1β, establishing a positive feedback loop between ROS and inflammatory signaling. Right: Within the BSCB, inflammatory cytokines and NF-κB reduce tight-junction proteins (occludin, claudin), upregulate ICAM-1/VCAM-1, and induce endothelial and astrocyte apoptosis, leading to barrier breakdown, increased permeability, leukocyte infiltration, and ultimately neurodegeneration with impaired repair.

In addition, ERS-mediated apoptosis poses a serious threat to BSCB integrity. In the harsh microenvironment following SCI, ERS persistently activates apoptotic pathways, leading to extensive apoptosis in BSCB constituent cells, especially endothelial cells and astrocytes (Feliziani et al., 2022). During apoptosis, cell membrane rupture and organelle release not only directly compromise the structural integrity of the BSCB but also induce stress responses in neighboring cells, further impairing BSCB function (Zheng et al., 2017). Furthermore, if cell debris and apoptotic bodies are not promptly cleared, they can act as secondary sources of damage by continuously stimulating inflammatory responses, thereby exacerbating BSCB injury and hindering nerve regeneration and functional recovery after injury (Zhao R. et al., 2022) (Figure 4).

## 6 The role of key molecules and signaling pathways in ERS-mediated cell death

### 6.1 PERK-eIF2α-ATF4-CHOP signaling pathway

The PERK-eIF2α-ATF4-CHOP signaling pathway is an important branch of the ERS response, serving as a key hub for the transition between cellular stress response and apoptosis, regulating the bidirectional choice of cell fate (Chen et al., 2022; Rozpedek et al., 2016). Under conditions of ERS, PERK is activated, leading to the phosphorylation of eIF2α, which results in overall inhibition of protein synthesis while promoting the translational upregulation of ATF4 (Akai et al., 2015). ATF4, as a transcription factor, induces the expression of the downstream target gene CHOP, an important pro-apoptotic factor whose upregulation usually indicates a transition of the cell towards the apoptotic program. This signaling pathway plays an important role in balancing the maintenance of cellular homeostasis and the response to sustained ERS, with excessive or prolonged activation promoting apoptosis (Moriguchi et al., 2019; Liu, 2020). Numerous studies have shown that the PERK-eIF2α-ATF4-CHOP pathway plays a key role in various disease states. For example, in a Lewis lung cancer mouse model, treatment induced cell cycle arrest and apoptosis by regulating the expression of proteins related to this pathway, effectively inhibiting tumor growth and demonstrating the potential of this pathway in anti-tumor therapy (Yang et al., 2025). Additionally, in a cardiomyocyte model, atractylenolide III alleviated ERS damage and apoptosis by inhibiting the GRP78/PERK/CHOP pathway, highlighting the significance of regulating this pathway for cardioprotection (Zuo et al., 2022). In neurological diseases, the PERK-eIF2α-ATF4-CHOP pathway is also critical; for instance, in the hippocampal neurons of aged mice, ERS, mediated by this pathway, is closely related to neuronal apoptosis, affecting postoperative cognitive dysfunction (POCD). The drug salubrinal, which inhibits this pathway, can improve cognitive function, suggesting its therapeutic potential (Wang et al., 2023). In chronic kidney failure models, activation of this pathway is believed to help alleviate hippocampal neuronal damage, indicating its complex regulatory role (Chen et al., 2023). In the field of tumors, the second-generation proteasome inhibitor Delanzomib induces ERS, activating the PERK-eIF2α-ATF4-CHOP pathway, which promotes cell cycle arrest and apoptosis in hepatocellular carcinoma cells, demonstrating its potential as an anti-tumor drug (Li et al., 2020c). Meanwhile, argininosuccinate synthase 1 (ASS1) inhibits tumor progression by activating this pathway, indicating its function in tumor suppression (Kim et al., 2021a). Among the intervention strategies targeting this pathway, PERK inhibitors such as GSK2656157 show neuroprotective potential. They significantly reduce the expression of ERS-related proteins and alleviate apoptosis. For example, GSK2656157 inhibited the activity of the PERK-eIF2α-ATF4-CHOP pathway and significantly reduced the apoptosis rate in a bisphenol A-induced TM4 cell apoptosis model (Lv et al., 2023; Liu S. et al., 2023). Similarly, in a polymyxin B-induced HK-2 cell injury model, the use of PERK inhibitors also reduced apoptosis, demonstrating their potential as therapeutic agents (Chen J. et al., 2024). Moreover, the activation of this pathway is not only related to traditional apoptosis but also involves various forms of cell death such as autophagy and ferroptosis, as well as processes like cell cycle regulation. For instance, in hepatocellular carcinoma and breast cancer cells, the regulation of the PERK-eIF2α-ATF4-CHOP pathway affects cell cycle arrest and apoptosis, and interacts with signaling pathways such as STAT3 and NF-κB, exhibiting a complex regulatory network (Li et al., 2020c; Zhang et al., 2021; Albayrak et al., 2021). In addition, this pathway is involved in the regulation of ferroptosis related to ERS by modulating the expression of antioxidant enzymes, thereby influencing the survival of neurons and cardiomyocytes (Lu et al., 2024; Liu C. et al., 2025). In summary, the PERK-eIF2α-ATF4-CHOP signaling pathway serves as a key hub for the transition between cellular stress and apoptosis, playing an important role in various diseases and pathological states.

### 6.2 The protective role and regulation of the IRE1α-XBP1 pathway

ERS is a common cellular response to various injuries. The IRE1α-XBP1 pathway, as the most conserved branch of the UPR, plays a key role in maintaining ER homeostasis. IRE1α is a transmembrane protein with kinase and ribonuclease activity. It senses the accumulation of misfolded proteins in the ER and activates its ribonuclease activity to splice XBP1 mRNA, producing the active spliced form of XBP1 (XBP1s). XBP1s acts as a transcription factor to promote the expression of various response genes, thereby facilitating protein folding, degradation, and recovery of ER function (Qiu et al., 2023). This process is crucial for maintaining ER homeostasis and enhances the cell’s ability to adapt to protein folding demands. In various tissues and cell types, the activity of the IRE1α-XBP1 pathway is significant for protecting cells from apoptosis induced by excessive ERS. For example, in a spinal motor neuron model, the Sig-1R agonist slows down motor neuron death by regulating the expression of IRE1α and XBP1. This suggests the potential role of this pathway in neuroprotection (Cao et al., 2019; Zhao et al., 2016). Additionally, in renal ischemia-reperfusion injury, the IRE1α-XBP1 signaling promotes autophagy and cell survival, which alleviates kidney injury (Liu et al., 2025b). In vascular smooth muscle cells, the activated IRE1α-XBP1 signaling pathway regulates cell phenotype switching and antioxidant responses, helping to inhibit the progression of vascular lesions (Huang et al., 2025; Zhang et al., 2025). These studies indicate that the IRE1α-XBP1 pathway plays an important role in protecting cell function by regulating protein folding, degradation, and cellular stress responses. The absence or dysfunction of XBP1 can exacerbate ERS responses, leading to cell death and tissue dysfunction (Qiu et al., 2023; Wiese et al., 2022). Mice with liver-specific XBP1 deletion exhibit exacerbated liver injury, inflammation, and fibrosis under a high-fructose diet. This is accompanied by excessive activation of IRE1α, indicating that the loss of XBP1 triggers dysregulation of IRE1α, thereby promoting liver injury (Duwaerts et al., 2021). Similarly, in diabetic nephropathy, CD248 induces an imbalance in adaptive UPR by inhibiting IRE1α-mediated XBP1 splicing, which promotes kidney injury (Krishnan et al., 2023). In an acute graft-versus-host disease model, specific deletion of XBP1 in intestinal epithelial cells leads to dysregulation of ERS signaling and exacerbates tissue damage. This suggests the critical impact of XBP1 on intestinal epithelial repair and tissue homeostasis (Haring et al., 2022). Furthermore, polymorphisms in OPTN in retinal cells are associated with abnormal XBP1 splicing, increasing sensitivity to ERS and promoting cell death (Sayyad et al., 2023). This evidence suggests that XBP1 deficiency weakens the protein folding capacity. It also induces excessive activation of IRE1α and terminal UPR signaling, leading to apoptosis and tissue damage. These effects severely impair the repair processes of the nervous system and other tissues. The regulation of the IRE1α-XBP1 pathway is complex and influenced by various molecules and signals. For instance, the neurotrophic factor MANF protects neurons from ERS damage by directly binding to IRE1α and inhibiting its excessive activation (Kovaleva et al., 2023). Additionally, the transcription factor PGRN can promote IRE1α phosphorylation and XBP1 mRNA splicing, maintaining ER homeostasis in chondrocytes and delaying the progression of osteoarthritis (Liang et al., 2023). In terms of pharmacological intervention, activators of IRE1α, such as Mn^2+^, enhance cell survival, whereas inhibitors, such as STF-083010, promote apoptosis. This suggests that regulating this pathway has therapeutic potential (Shi et al., 2025; Barez et al., 2020). Moreover, epigenetic modifications also participate in the regulation of this pathway, as the methylation status of H3K9 and H3K27 affects XBP1 gene expression and regulates ERS responses (Nguyen et al., 2022). In summary, the IRE1α-XBP1 pathway protects cells by promoting protein folding and degradation, thereby maintaining ER homeostasis. XBP1 deficiency leads to dysregulation of ERS signaling, promoting cell death and tissue damage, which adversely affects the repair processes of the nervous system and other tissues.

### 6.3 AMPK-mTOR signaling and autophagy activation

5′-adenosine monophosphate-activated protein kinase (AMPK) and mTOR (mammalian target of rapamycin) signaling pathways play a central role in the regulation of cellular autophagy (Tang B. et al., 2023). AMPK, as an energy sensor, can be activated when cellular energy is deficient, thereby promoting autophagy to maintain cellular homeostasis, whereas mTOR acts as an inhibitor of autophagy. When mTOR activity decreases, autophagy is activated. The reciprocal regulation of these two signaling pathways is crucial for modulating the cellular response to ERS (Rui et al., 2015). ERS contributes significantly to neuronal injury following SCI by inducing cell death. Regulating autophagy mediated by the AMPK-mTOR pathway may therefore become an important strategy to alleviate ERS and protect neurons (Bisicchia et al., 2022).

Sestrin2 is a stress-induced protein that promotes autophagy by activating the AMPK signaling pathway, thereby alleviating ERS and oxidative stress, and exerting neuroprotective effects. Studies have shown that upregulation of Sestrin2 activates AMPK, which inhibits mTOR activity and initiates the autophagy process. This leads to the clearance of damaged proteins and organelles, reducing the cellular stress burden (Lee et al., 2025; Ala, 2023). For example, in various disease models, Sestrin2-mediated AMPK activation promotes autophagy, reduces neuronal apoptosis and inflammatory reactions, and enhances cell survival. This mechanism also applies in the context of SCI, where enhancing Sestrin2 expression or its downstream AMPK activity effectively activates autophagy, alleviates ERS-induced neuronal damage, and promotes neuronal survival and functional recovery (Li et al., 2021; Scalabrin et al., 2022).

Transcription factor E3 (TFE3), a member of the MiT family, acts as a transcriptional regulator of autophagy and lysosomal genes, participating in the regulation of intracellular autophagy processes (Kim et al., 2021b). TFE3 can sense intracellular ROS levels and ERS status, coordinating the clearance of damaged proteins and oxidative damage by regulating the expression of autophagy-related genes (Foerster et al., 2022). Research has found that TFE3 activity is regulated by the AMPK-mTOR signaling pathway. Specifically, AMPK activation promotes TFE3 nuclear translocation, which enhances the transcription of autophagy genes, reduces ROS accumulation, and alleviates ERS. Dysregulation of TFE3 function can lead to elevated ROS levels and exacerbated ERS, promoting neuronal apoptosis. In SCI, TFE3 helps clear ROS and abnormal proteins within damaged cells by regulating autophagy activity mediated by the AMPK-mTOR signaling pathway, thereby alleviating ERS and protecting neurons from further damage (Yang et al., 2022; Chen et al., 2025; Paquette et al., 2021). In summary, the AMPK-mTOR signaling pathway mediates autophagy activation through the regulation of key factors such as Sestrin2 and TFE3, playing an important role in alleviating ERS and oxidative stress, and promoting neuroprotection. Regulating this signaling axis not only helps maintain cellular homeostasis but also provides potential therapeutic targets for the protection and repair of neurons after SCI.

### 6.4 Other regulatory factors and pathways

In the process of SCI, in addition to the classical ERS signaling pathways, an increasing number of studies focus on the effects of other regulatory factors and signaling pathways on ERS and cell fate, especially the regulatory role of the Sigma-1 receptor and the interactions between oxidative stress and inflammatory signals with ERS (Hayashi, 2019). These factors not only regulate cell survival and death but also have significant implications for the protection of motor neurons and the recovery of spinal cord function. First, the Sigma-1 receptor (Sigma-1R), as an endoplasmic reticulum-associated molecule, plays a key role in the protection of motor neurons (Mancuso and Navarro, 2017). Sigma-1R is highly expressed in motor neurons, and its agonists, such as PRE-084, SA4503, and BD1063, have shown significant neuroprotective effects in spinal nerve root injury models. Mechanistically, Sigma-1R alleviates the ERS response by regulating the ERS markers IRE1α and XBP1, thereby reducing the death of motor neurons. This mechanism has been confirmed to be absent in Sigma-1R knockout mice, indicating that Sigma-1R is a key regulatory factor for motor neuron survival. By activating Sigma-1R-related signaling pathways, it is possible to effectively reduce neuronal apoptosis after SCI and promote tissue repair and functional recovery. These findings demonstrate its potential as a therapeutic target (Tsai et al., 2009). Secondly, oxidative stress and inflammatory signals are closely intertwined with ERS, jointly influencing cell fate after SCI. After SCI, ROS levels significantly increase, leading to exacerbated oxidative stress. This further impairs the autophagic function of cells, resulting in autophagic flux blockage and causing the accumulation of intracellular proteins and damaged organelles, which reinforces the ERS state. The reinforced ERS state, in turn, promotes the release of inflammatory mediators, exacerbating cell damage and apoptosis (Zhou et al., 2020; Yin et al., 2024; Fassbender et al., 2012). For example, transcription factor E3 (TFE3) has been found to regulate ROS-mediated autophagic dysfunction, alleviate ERS and promote neuronal survival. TFE3 improves autophagic flux and reduces ROS levels by activating the AMPK-mTOR and AMPK-SKP2-CARM1 signaling pathways, thereby alleviating ERS-induced neuronal death and enhancing functional recovery (Yang et al., 2022; Lou et al., 2022).

**FIGURE 5:**
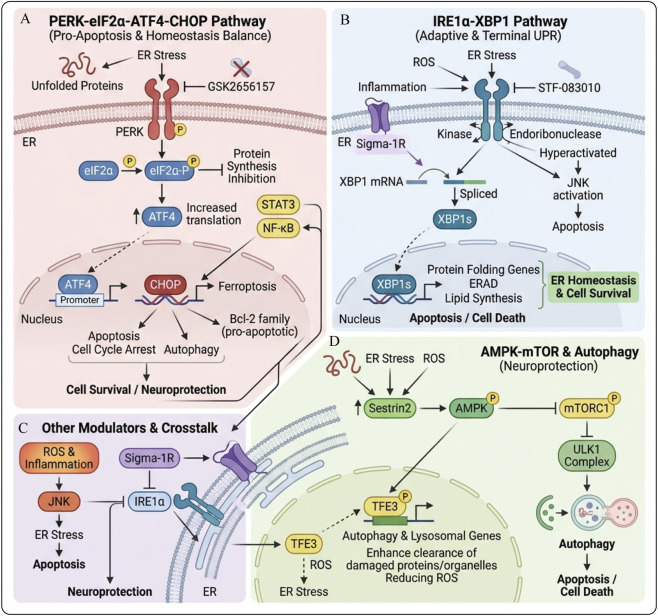
ERS–mediated cell fate decisions and modulatory pathways **(A)**. The PERK–eIF2α–ATF4–CHOP pathway is activated by unfolded proteins, suppresses global translation, upregulates ATF4/CHOP, and triggers apoptosis, autophagy and ferroptosis, while integrating with STAT3 and NF-κB survival/inflammatory signals **(B)**. The IRE1α–XBP1 branch promotes protein folding and ERAD via XBP1s to maintain ER homeostasis under adaptive stress, but switches to JNK-driven apoptosis when hyperactivated **(C)**. The Sestrin2–AMPK–mTOR axis inhibits mTORC1, enhances autophagy and lysosomal gene expression, clears damaged proteins/organelles and lowers ROS, providing neuroprotection **(D)**. Additional modulators such as Sigma-1R, JNK and IRE1α couple ROS/inflammation to ER stress, either amplifying cell death or conferring protection, and offer potential therapeutic targets within the ER stress–autophagy–inflammation network.

In addition, oxidative stress also participates in the regulation of ERS by modulating Sestrin2 expression. Sestrin2 is a stress-induced metabolic regulatory protein that can activate the Nrf2 and AMPK signaling pathways and negatively regulate mTORC1; thereby promoting autophagy, alleviating ERS and oxidative damage, reducing apoptosis and inflammatory responses, and enhancing neuronal survival and functional recovery (Ala, 2023; Ala and Eftekhar, 2021; Gong et al., 2021). Regarding inflammatory signals, astrocytes in the spinal cord and brainstem exhibit region-specific reactive states during neuroinflammation, with their activation closely related to ERS. Inflammatory mediators such as IL-1α, TNF-α, and IL-6 can induce a reactive phenotype in astrocytes, activate the JNK signaling pathway, and enhance the ERS response. These effects produce neurotoxicity and promote motor neuron death (Lindblad et al., 2023). Similarly, in studies on amyotrophic lateral sclerosis (ALS), inflammatory responses and ERS synergistically promote the inflammatory state of astrocytes and inhibit their ability to support neurons, thereby exacerbating neuronal damage; this suggests that inflammation-mediated ERS is an important pathological mechanism in neurodegenerative diseases (Sun et al., 2015; Ziff et al., 2022). In summary, the Sigma-1 receptor exerts a protective effect on motor neurons by regulating ERS-related signals and provides new therapeutic targets for neuroprotection after SCI (Figure 5).

## 7 Treatment strategies for ERS for SCI

**FIGURE 6:**
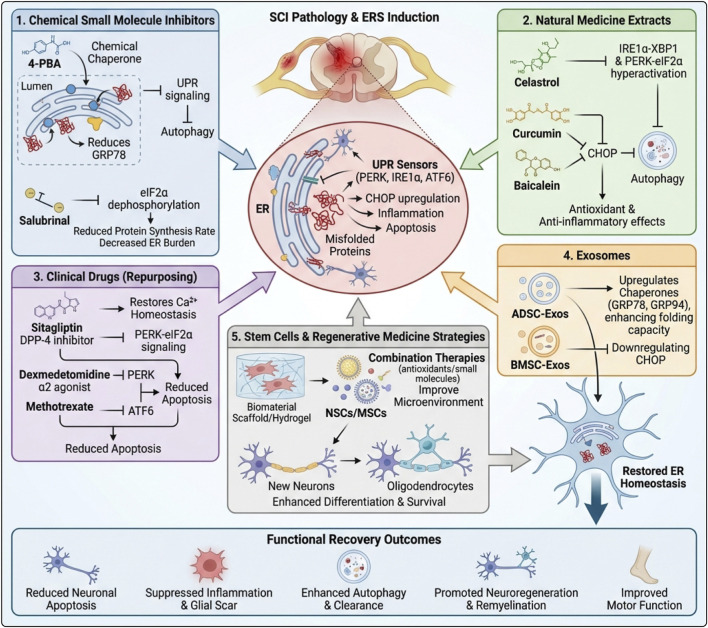
Multidimensional therapeutic strategies targeting ERS to promote SCI repair. Centrally, SCI triggers accumulation of misfolded proteins and activates UPR sensors (PERK, IRE1α, ATF6), leading to CHOP upregulation, inflammation, and apoptosis. (1) shows chemical small-molecule inhibitors such as 4-PBA and Salubrinal acting as chemical chaperones and modulating eIF2α phosphorylation to reduce ER burden. (2) illustrates natural medicine extracts (celastrol, curcumin, baicalein) that fine-tune IRE1α–XBP1 and PERK–eIF2α pathways, suppress CHOP, induce autophagy, and exert antioxidant/anti-inflammatory effects. (3) highlights repurposed clinical drugs (sitagliptin, dexmedetomidine, methotrexate) that restore Ca^2+^ homeostasis, modulate UPR signaling, and attenuate apoptosis. (4) depicts stem cell–derived exosomes (ADSC-Exos, BMSC-Exos) upregulating chaperones (GRP78, GRP94), downregulating CHOP, and enhancing protein-folding capacity. (5) outlines stem cell and regenerative medicine approaches combining NSCs/MSCs with antioxidants/small molecules and biomaterial scaffolds to improve the microenvironment and support neuron/oligodendrocyte differentiation and survival.

In secondary injury of SCI, ERS and its UPR play a compensatory protective role in the early stage. However, sustained activation can disrupt protein homeostasis through the PERK–eIF2α–ATF4/CHOP, IRE1α–XBP1, and ATF6 axes, thereby amplifying the inflammatory response and inducing neuronal cell death. Given the close coupling of UPR with autophagy, mitochondrial dysfunction, oxidative stress, and apoptosis/pyroptosis pathways, targeting ERS to restore protein homeostasis offers a promising therapeutic approach for neuroprotection and repair in SCI. Existing intervention strategies include small-molecule chemicals directly regulating ERS/UPR, natural drug components with multi-target effects, repositioning of clinically available drugs, and biological therapy schemes such as exosomes and stem cell-based regenerative medicine (Figure 6).

### 7.1 Chemical small molecule inhibitors

Chemical small molecule inhibitors have shown significant potential in regulating ERS and improving SCI. 4-phenylbutyric acid (4-PBA), as a well-established ERS inhibitor, primarily acts by binding to molecular chaperones in the endoplasmic reticulum lumen. This binding indirectly regulates the UPR signaling pathway and alleviates the degree of ERS (Wang et al., 2024). In animal models of SCI, researchers found that after treatment with 4-PBA, the expression levels of ERS marker proteins, such as GRP78: in spinal cord tissue were significantly reduced, indicating effective relief of ERS. Meanwhile, the number of neuronal apoptosis significantly decreased, and the inflammatory response in the SCI area was also significantly suppressed, reducing inflammation and apoptosis, which in turn facilitates neurological recovery (Mizukami et al., 2010). Additionally, *in vitro* experiments have found that 4-PBA can enhance autophagy by inhibiting ERS, thereby alleviating damage (Wang et al., 2020).

Salubrinal is another potent ERS inhibitor that can specifically inhibit the dephosphorylation process of eIF2α, thereby regulating the rate of protein synthesis and reducing the protein folding load on the endoplasmic reticulum (Nguyen et al., 2024). Experimental studies have shown that timely intervention with Salubrinal after SCI can effectively inhibit the ERS-mediated apoptosis pathway, thus reducing neuronal loss. Long-term observation of rats with SCI revealed that the Salubrinal treatment group exhibited a significantly accelerated recovery process of neurological function, and the degree of pathological damage in their spinal cord tissue was also significantly reduced, providing strong experimental evidence for the application of Salubrinal in clinical treatment of SCI (Ohri et al., 2013). Despite these encouraging results of chemical small molecule inhibitors in animal experiments, numerous challenges remain in clinical applications, such as drug safety, optimal dosing, and optimal timing of administration. Therefore, further in-depth research is required to address these issues.

### 7.2 Natural medicinal extract

Natural drug extracts, with their multi-target and low-toxicity advantages, have opened new avenues for the treatment of SCI. Currently, various natural drug extracts have been confirmed to exhibit unique potential in regulating ERS and promoting nerve repair. Celastrol, an active component extracted from the traditional Chinese medicine Tripterygium wilfordii, possesses biological activities such as antioxidation, anti-inflammation, and regulation of ERS (Schiavone et al., 2021). Research has found that Celastrol can reduce the occurrence of apoptosis by inhibiting the excessive activation of the ERS signaling pathways, such as the IRE1α-XBP1 and PERK-eIF2α pathways (Liu DD. et al., 2020). In the SCI rat model, after treatment with Celastrol, the levels of inflammatory factors in the spinal cord tissue of rats significantly decreased, the number of surviving neurons increased markedly, and motor function also improved to some extent (Shen et al., 2024). However, the clinical application of Celastrol is limited by its potential hepatotoxicity. Therefore, optimizing its dosage and formulation to reduce toxicity has become a major focus of current research.

Curcumin, a natural polyphenolic compound extracted from turmeric rhizomes, has powerful antioxidative, anti-inflammatory, and neuroprotective effects (Tabrizi et al., 2019; Ghandadi and Sahebkar, 2017; Wu et al., 2006). Similarly, recent studies have shown that curcumin can alleviate secondary injury after SCI by regulating specific ERS-related signaling pathways (Zhu et al., 2017). Curcumin can inhibit ERS induced apoptosis and promote moderate activation of autophagy, helping to clear damaged organelles and protein aggregates; thus, it provides a favorable environment for cell repair and regeneration. *In vitro* cell experiments have shown that curcumin pretreatment can significantly improve the survival rate of spinal cord neurons under oxidative stress conditions and reduce the expression of ERS markers. *In vivo* experiments have also confirmed that curcumin treatment can improve hind limb motor function in rats with SCI and reduce scar formation at the injury site, thereby creating favorable conditions for nerve regeneration (Chen T. et al., 2024). Although curcumin has a high safety profile, issues such as low bioavailability limit its clinical efficacy.

Baicalin, one of the main components of the traditional Chinese medicine Scutellaria baicalensis, possesses various biological activities, such as anti-inflammatory and anti-oxidation (Li et al., 2018b). Baicalin may inhibit ERS (ERS)-induced apoptosis by suppressing the CHOP pathway (Choi et al., 2010). Studies have found that in the SCI mouse model, baicalin enhances functional recovery by activating autophagy to clear unfolded proteins and damaged mitochondria, thereby reducing ERS-mediated apoptosis and inhibiting pyroptosis (Wu et al., 2020). In addition to these natural product extracts, icariin (Li H. et al., 2019), ginsenoside (Dou et al., 2018), and resveratrol (Tian et al., 2024) similarly promote the recovery of neurological function after injury by inhibiting ERS-induced neuronal apoptosis following SCI. These natural product extracts offer new ideas and directions for the treatment of SCI; however, achieving their clinical translation requires overcoming many technical challenges, such as optimizing delivery methods, ensuring bioavailability, and confirming safety and efficacy in humans. Therefore, it is necessary to conduct in-depth basic research and clinical trials to fully explore their therapeutic potential and provide more treatment options for patients with SCI.

### 7.3 Clinical drug

In addition to chemical small molecule inhibitors and natural drug extracts, some commonly used clinical drugs have also shown potential therapeutic value in the treatment of ERS in SCI. These drugs regulate ERS-related signaling pathways through different mechanisms of action, providing new therapeutic approaches for the treatment of SCI.

Sitagliptin is a selective dipeptidyl peptidase-4 inhibitor with antioxidant, anti-inflammatory, and anti-apoptotic effects; it is primarily used in clinical practice for the treatment of type 2 diabetes mellitus (Karasik et al., 2008). Studies have found that Sitagliptin can reduce neuronal apoptosis by restoring cellular calcium homeostasis and inhibiting ERS (Kizilay et al., 2021). Recent studies have found that Sitagliptin also has promising potential in the treatment of SCI (Han et al., 2020). In animal models of spinal cord injury, treatment with Sitagliptin significantly reduced the expression levels of ERS-related proteins GRP78 and CHOP. Additionally, the number of apoptotic neurons was significantly decreased. Further mechanistic studies indicate that Sitagliptin may reduce the accumulation of unfolded or misfolded proteins by inhibiting the activation of the PERK-eIF2α pathway, thereby alleviating ERS-induced neuronal damage and promoting the regeneration of the injured spinal cord (Tang C. et al., 2023).

Dexmedetomidine is an α2 adrenergic receptor agonist with biological effects such as sedation, analgesia, anti-anxiety, and sympathetic inhibition, widely used in clinical practice (Mahmoud and Mason, 2015). Studies have found that Dexmedetomidine exhibits neuroprotective effects in various models of nerve injury, including spinal cord injury. For example, Dexmedetomidine can improve neurological function in rats with traumatic spinal cord injury and reduce spinal cord neuron apoptosis (Liu B. et al., 2023; Liu MJ. et al., 2022). In spinal cord injury research, Dexmedetomidine has been found to regulate ERS-related signaling pathways, reducing the degree of ERS to decrease spinal cord neuron apoptosis, thereby improving neurological functional recovery after injury (Liu YB. et al., 2022).

Methotrexate is an anti-metabolite drug with anti-inflammatory and immunosuppressive effects (Fleischmann et al., 2017). It has been reported that low-dose Methotrexate used for SCI treatment can reduce inflammation, oxidative stress, and apoptosis, thereby preventing the occurrence and development of secondary injury (Sanli et al., 2012; Bakar et al., 2013; Kertmen et al., 2013). Recent studies have found that Methotrexate also has a regulatory effect on ERS in spinal cord injury. In in vivo and *in vitro* experiments, Methotrexate was found to inhibit the expression of ERS-related proteins by suppressing the activation of the PERK-eIF2α pathway and the ATF6 pathway, thereby inhibiting ERS-induced apoptosis and promoting the recovery of damaged neurological function (Rong et al., 2018). Although these drugs have been widely used in clinical practice, they are still in the preclinical stage in the field of SCI. To achieve clinical translation, many technical challenges still need to be overcome. Therefore, it is necessary to conduct in-depth basic research and clinical trials to fully explore their therapeutic potential and assess their feasibility and safety.

### 7.4 Exosomes

Exosomes, as nano-sized vesicles secreted by cells, contain various bioactive molecules such as proteins, nucleic acids, and lipids, playing an important role in cell communication (Chen et al., 2021; Fang et al., 2020). In recent years, the potential of exosomes in SCI treatment has gradually attracted attention, especially their role in regulating ERS, offering novel therapeutic approaches for SCI (Yao et al., 2023). Research shows that ADSC-derived exosomes can regulate endoplasmic reticulum homeostasis in damaged nerve cells by delivering specific proteins and nucleic acid molecules. This process promotes the survival and regeneration of nerve cells (Zhang et al., 2024). Specifically, ADSC-derived exosomes alleviate nerve cell damage by modulating ERS-related signaling pathways. Studies have reported that these exosomes upregulate the expression of molecular chaperones in the endoplasmic reticulum, such as GRP78 and GRP94; this enhances the protein folding capacity of the ER and reduces the accumulation of unfolded or misfolded proteins, thereby alleviating ERS (Lu et al., 2022). In addition, exosomes from BMSCs also play a protective role in SCI. Injection of BMSC-derived exosomes into SCI rat models, as a therapeutic intervention, reduces the expression of CHOP in injured motor neurons and inhibits ERS, thereby alleviating neuronal apoptosis and inflammatory responses in SCI rats (He et al., 2022b; Gu et al., 2017).

### 7.5 Stem cells and regenerative medicine strategies

In recent years, stem cell therapy and regenerative medicine have received significant attention in the application of SCI repair. After SCI, a large number of neural cells die and axons break, leading to functional impairment. Endogenous and transplanted stem cells participate in nerve regeneration and myelin repair by differentiating into neurons and oligodendrocytes, demonstrating significant therapeutic potential. Current research focuses on regulating the survival, proliferation, and directed differentiation of stem cells, optimizing the microenvironment, and enhancing therapeutic efficacy (Karimi-Abdolrezaee et al., 2006).

Neural stem cells (NSCs), as multipotent stem cells, also play a key role in SCI repair. NSC-based therapeutic strategies reshape the neural network by promoting cell proliferation and inducing differentiation into neurons and oligodendrocytes (Guo et al., 2022). For example, application of IGF-1-loaded bioactive nanohydrogels can activate downstream signals of IGF-1, prevent NSC apoptosis, promote their proliferation and differentiation into neurons and oligodendrocytes, ultimately facilitating axon growth and myelin regeneration (Song et al., 2024). Furthermore, electroactive scaffolds combined with NSCs can continuously stimulate the paracrine function of NSCs, alleviate oxidative stress and inflammation, inhibit neuronal cell death, and promote neurogenesis and functional neural circuit restoration (Liu et al., 2025c). These studies emphasize the molecular mechanisms regulating the biological characteristics of stem cells, particularly how ERS influences cell differentiation, providing a theoretical basis for designing effective stem cell therapy strategies.

Because the microenvironment after SCI is characterized by intense inflammation and oxidative stress, the efficacy of single stem cell therapy is limited. Therefore, combined drug therapy strategies have been widely explored to enhance the effects of stem cell therapy. For instance, delivering antioxidants, anti-inflammatory drugs, or small molecules that regulate cell death pathways via nanocarriers can improve the survival and differentiation efficiency of stem cells (Tsakiris et al., 2020; Zibara et al., 2019). In research, the use of a composite drug containing four small molecules (LDN193189, SB431542, CHIR99021, P7C3-A20), injected in combination with collagen hydrogel, successfully induced endogenous NSCs to differentiate into neurons, reduced astrocyte formation, and promoted nerve regeneration and recovery of motor function (Yang et al., 2021). Additionally, combining stem cell transplantation with targeted anti-inflammatory drug therapy has achieved improved outcomes, such as reduced inflammation and enhanced functional recovery. For example, intravenous injection of NE-4C neural stem cells can reduce the expression of L-selectin, decrease the inflammatory response at the site of SCI, and promote the survival and functional reconnection of motor neurons (Bellák et al., 2025).

Similarly, the synergistic treatment combining nanocarrier delivery of ferroptosis inhibitors with mesenchymal stem cells significantly inhibited inflammation and cell death, thereby promoting the recovery of neural function (Hua et al., 2024). These strategies demonstrate that jointly regulating the injured microenvironment with drugs and enhancing the survival and functional capabilities of stem cells are key to improving the efficacy of SCI treatment. Meanwhile, modifying stem cells or their exosomes with functionalized nanomaterials to enhance targeting and bioactivity has become a current research hotspot. For instance, exosomes derived from M2 macrophages and modified with the IKVAV peptide can target the injured site, promote stem cell neural differentiation, and suppress inflammation, thereby significantly improving motor function (Zeng et al., 2023). Additionally, conductive hydrogel carriers delivering brain-derived neurotrophic factor (BDNF) promote NSCs to differentiate into neurons and inhibit the generation of astrocytes, improving the structural integrity of the injured tissue (Huang F. et al., 2021). These novel combination therapy strategies enhance the safety and efficacy of cell therapy through multi-target and multi-pathway synergistic actions, providing a solid foundation for future clinical applications (Table 1).

**TABLE 1:** Potential regulatory modes and regulators of ERS in SCI treatment.

Strategy category	Intervention/Drug (source)	Main ERS-related targets/Mechanisms	Main effects	References
Chemical small molecule inhibitors	4-Phenylbutyric Acid (4-PBA)	Binds to ER lumen molecular chaperones, indirectly regulates UPR, significantly downregulates GRP78; inhibits ERS and enhances autophagy	Reduces ERS, decreases neuronal apoptosis and inflammatory response, improves neurological function recovery	Wang et al. (2020)
	Salubrinal	Specifically inhibits eIF2α dephosphorylation, alleviates protein folding burden, relieves excessive activation of the PERK-eIF2α-ATF4 pathway	Inhibits ERS-mediated apoptosis, reduces neuronal loss, alleviates tissue pathological damage, accelerates functional recovery	Ohri et al. (2013)
Natural compound	Tripterygium Wilfordii Glycosides	Inhibits excessive activation of ERS pathways such as IRE1α-XBP1, PERK-eIF2α	Antioxidant, anti-inflammatory, reduces apoptosis, lowers inflammation factors, increases neuronal survival, improves motor function; potential hepatotoxicity exists	(Liu et al., 2020b; Shen et al.)
	Curcumin	Regulates ERS-related signals; inhibits ERS-induced apoptosis; moderately activates autophagy to clear damaged organelles and protein aggregates	Increases neuronal survival under oxidative stress, downregulates ERS markers; can improve hind limb motor function and reduce scar formation *in vivo*; limited by low bioavailability	(Chen et al., 2024b)
	Baicalein	Might alleviate ERS-induced apoptosis by inhibiting the CHOP pathway; activates autophagy to clear misfolded proteins and damaged mitochondria	Reduces ERS-related apoptosis and pyroptosis, promotes functional recovery in SCI mice	Wu et al. (2020)
	Icariin	Inhibits ERS-induced neuronal apoptosis after SCI	Promotes neurological function recovery	Li et al. (2019b)
	Panax saponins	Inhibits ERS-related apoptosis pathways	Improves neurological function after injury, exerts neuroprotective effects	Dou et al. (2018)
	Resveratrol	Reduces ERS-induced neuronal apoptosis and oxidative stress	Promotes motor function recovery and neural repair	Tian et al. (2024)
Clinical drug	Sitagliptin	Restores cellular calcium homeostasis; inhibits PERK-eIF2α pathway activation, downregulates GRP78, CHOP	Antioxidant stress, anti-inflammatory, anti-apoptotic; reduces neuronal cell death, promotes regeneration of injured spinal cord	Han et al. (2020), Tang et al. (2023b)
	Dexmedetomidine	Regulates ERS-related signaling pathways, alleviates the degree of ERS	Reduces neuronal apoptosis and improves neurological function in various nerve injury models; alleviates ERS-related neuronal damage in SCI	Liu et al. (2023b), Liu et al. (2022a)
	Methotrexate	Inhibits activation of the PERK-eIF2α and ATF6 pathways, downregulates ERS-related proteins	Anti-inflammatory and immunosuppressive; alleviates inflammation, oxidative stress, and ERS-induced apoptosis, prevents progression of secondary injury, promotes functional recovery	Sanli et al. (2012), Bakar et al. (2013), Kertmen et al. (2013)
Exosomes	ADSCs-Exos	Upregulates expression of molecular chaperones such as GRP78, GRP94, enhances protein folding capacity, reduces accumulation of misfolded/incorrectly folded proteins	Alleviates ERS, reduces neuronal damage, promotes neuronal cell survival and regeneration	Zhang et al. (2024), Lu et al. (2022)
	BMSCs-Exos	Downregulates CHOP expression in injured motor neurons, inhibits ERS	Reduces neuronal apoptosis and inflammatory response, exerts significant neuroprotective effects	He et al. (2022b), Gu et al. (2017)
Stem cells/Regenerative medicine	NSCs + IGF-1 Bioactive Nanohydrogel	Activates IGF-1 downstream signals, prevents NSCs apoptosis, improves inflammation/oxidative stress and ERS-related microenvironment	Promotes NSCs proliferation and differentiation into neurons and oligodendrocytes, promotes axon growth and myelin regeneration	Guo et al. (2022), Song et al. (2024)
	Electroactive Scaffold + NSCs	Continuously stimulates NSCs paracrine, alleviates oxidative stress and inflammation, synergizes with ERS regulation	Inhibits neuronal death, promotes neurogenesis and neural circuit reconstruction	Liu et al. (2025c)
	Four Small Molecules (LDN193189, SB431542, CHIR99021, P7C3-A20) + Collagen Hydrogel	Regulates endogenous NSCs fate and injury microenvironment through multi-target small molecules, indirectly affects ERS levels	Induces endogenous NSCs differentiation into neurons, reduces astrocyte formation, promotes nerve regeneration and motor function recovery	Yang et al. (2021)
	NE-4C Neural Stem Cell Intravenous Injection	Reduces L-selectin expression, alleviates inflammation and oxidative stress, indirectly improves ERS-related microenvironment	Promotes motor neuron survival and functional reconnection, improves functional recovery	Bellák et al. (2025)
	Iron Death Inhibitors + MSCs (Combined Nanocarrier Delivery)	Inhibits iron death pathway and its interaction with ERS/oxidative stress; improves stem cell survival environment	Significantly inhibits inflammation and cell death, promotes neurological function recovery	Bellák et al. (2025)
	IKVAV Peptide Modified M2 Macrophage-Derived Exosomes	Increases exosome targeting to injured spinal cord; promotes stem cell neural differentiation and inhibits inflammation, synergizes with ERS regulation	Significantly improves motor function, enhances nerve regeneration	Zeng et al. (2023)
	Conductive Hydrogel Carrier Delivery BDNF + NSCs	Conductive scaffold + BDNF jointly regulates NSCs fate, improves inflammation/oxidative stress and ERS-related microenvironment	Promotes NSCs differentiation into neurons, inhibits astrocyte generation, improves injured tissue structure and neural network reconstruction	Huang et al. (2021c)

## 8 Future research directions

In the process of SCI, ERS exhibits complex and dynamic changes, with significant differences in its manifestation across various cell types. Previous studies have utilized single-cell RNA sequencing (scRNA-seq) combined with bulk RNA sequencing methods to reveal the critical role of oligodendrocyte precursor cells in ERS activation and subsequent differentiation after injury. These studies found that OPCs significantly activate ERS after SCI, and inhibiting ERS can effectively reduce OPC death, suggesting that ERS has varying impacts on the survival and function of OPCs at different stages of injury and differentiation (Zhao Q. et al., 2022). Furthermore, research has shown that specific deletion of the XBP1 gene in oligodendrocytes exacerbates the ERS response, limiting motor function recovery and white matter protection, further demonstrating that the dynamic regulation of ERS in oligodendrocytes is crucial for SCI recovery (Saraswat et al., 2021). Future research should focus on utilizing high-throughput single-cell omics technologies at multiple time points and across various cell types to systematically depict the spatiotemporal dynamics of ERS signaling pathways after SCI. This approach will clarify the activation levels of ERS markers and the duration of ERS in different cell types, such as neurons, oligodendrocytes, astrocytes, and microglia. Additionally, combining transcriptomic, proteomic, and metabolomic data is expected to elucidate the molecular mechanisms of ERS at various stages and their relationship with cell fate decisions, promoting the development of precise intervention strategies.

**FIGURE 7:**
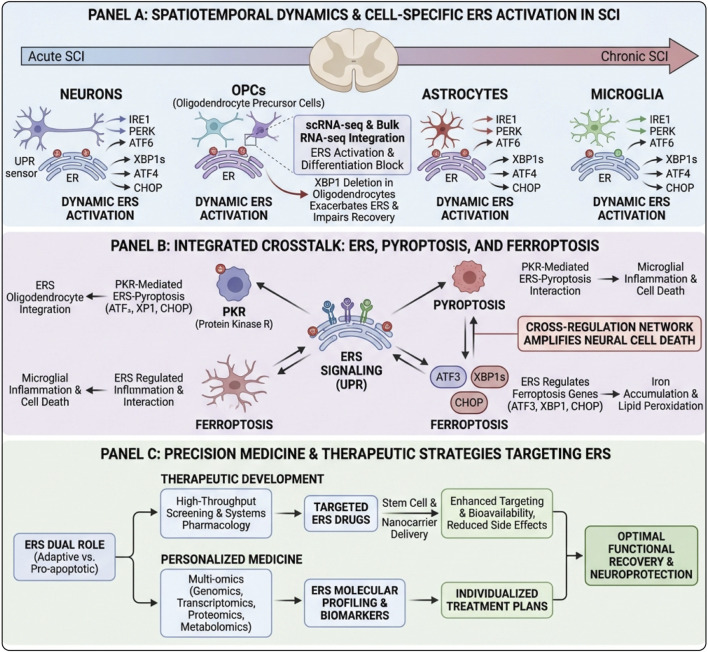
Dynamic activation of ERS in SCI, its interaction with cell death, and precise intervention ideas **(A)**. From acute to chronic SCI, neurons, OPCs, astrocytes and microglia exhibit dynamic activation of IRE1/PERK/ATF6 and downstream XBP1s, ATF4, CHOP; excessive ERS impairs oligodendrocyte differentiation and functional recovery **(B)**. ERS crosstalk with pyroptosis and ferroptosis via PKR and ERS-driven transcription promotes inflammation, iron accumulation and lipid peroxidation, amplifying neural cell death **(C)**. Recognizing the dual adaptive vs. pro-apoptotic roles of ERS, multi-omics profiling and biomarkers can support development of ERS-targeted drugs and personalized treatment plans to optimize neuroprotection and functional recovery.

In recent years, in addition to traditional apoptosis, new forms of programmed cell death such as pyroptosis and ferroptosis have gradually gained attention in the pathological mechanisms of SCI. Existing studies have shown that PKR (protein kinase R) interacts with ERS and pyroptosis pathways in SCI, mediating the inflammatory response and cell death of microglia. Inhibiting PKR can alleviate ERS and pyroptosis, thereby promoting functional recovery (Yang et al., 2024). Moreover, ferroptosis is closely associated with ERS. Through bioinformatic analysis, it was found that ferroptosis-related genes, including ATF3, XBP1 (which is a key factor in ERS), and CHOP, are significantly upregulated after SCI, indicating that ERS signaling plays an important role in the regulation of ferroptosis (Kang et al., 2022). These results suggest that ERS not only independently induces cell apoptosis but also cross-regulates the cell death pathways of pyroptosis and ferroptosis, exacerbating neuronal cell death in SCI. Future research should focus on revealing the cross-regulatory network between ERS and key molecules involved in pyroptosis and ferroptosis. It is especially important to clarify the mechanisms of action in different cell types and stages of injury. Combining gene editing and drug intervention strategies will help clarify the interactions among various forms of cell death. This will provide a theoretical basis for the comprehensive treatment of SCI. Given the complex bidirectional regulatory role of ERS in SCI, precise regulation of ERS for personalized treatment has become a key focus of future research. Multiple studies have validated the potential of various drugs and interventions in regulating ERS and promoting neuroprotective effects (Saraswat et al., 2023; Zhu et al., 2020). Building on these findings, future efforts should combine high-throughput screening and systems pharmacology approaches to develop targeted drugs for specific ERS signaling pathways. Additionally, utilizing genomic, epigenetic, and metabolomic biomarkers to construct a molecular profile of ERS in SCI patients will enable individualized and precise treatment plans. At the same time, integrating stem cell technology and nanocarrier systems can enhance the targeting and bioavailability of therapeutic drugs, reduce side effects, and promote functional recovery and improve quality of life for SCI patients (Figure 7).

## 9 Conclusion

ERS plays a dual role in the pathophysiological process after SCI, serving as an important regulatory factor for cell protection and repair, while also being a key mechanism that triggers PCD. Through a comprehensive analysis of existing studies, it is evident that the activation of ERS-related signaling pathways has a decisive impact on the determination of the fate of neural cells after SCI. Moreover, the complexity and diversity of its regulation reflect the intricate network of SCI’s pathological mechanisms. It is essential to examine the development and clinical potential of this mechanism from a multidimensional perspective, integrating differing findings on the protective and lethal effects of ERS in various studies to promote the rational design and optimization of management strategies. In the future, through interdisciplinary collaboration and technological innovation, precise regulation of ERS-related pathways is expected to become an important strategy for improving the prognosis and functional recovery of SCI patients, having a profound impact on the advancement of the field of neural repair.
